# Party identification and public attitudes toward raw milk, water fluoridation, and acetaminophen use

**DOI:** 10.1093/haschl/qxag237

**Published:** 2026-09-24

**Authors:** Filip Viskupič, David L Wiltse, Md Shahriar Mahmud, Claus Kadelka

**Affiliations:** Department of Political Science, Iowa State University, Ames, IA, USA; School of American and Global Studies, South Dakota State University, Brookings, SD, USA; Department of Mathematics, Iowa State University, Ames, IA, USA; Department of Mathematics, Iowa State University, Ames, IA, USA

**Keywords:** water fluoridation, raw milk, acetaminophen, public opinion, political partisanship

## Abstract

**Background:**

Fluoridation of drinking water, milk pasteurization, and acetaminophen use in pregnancy have become highly visible and controversial public health issues.

**Methods:**

In September and October 2025, we fielded a national survey to examine the beliefs about and support for policies regulating water fluoridation, raw milk, and acetaminophen use in the United States. We used descriptive statistics and multivariate logistic regression to analyze the data.

**Results:**

We found that about half of the participants believed fluoridated tap water was safe to drink, that raw milk was not as safe to drink as pasteurized milk, and that taking acetaminophen during pregnancy did not cause autism. About a third of respondents reported that water fluoridation should be banned, sale of raw milk should be liberalized, and that the government should issue new guidelines against the use of acetaminophen during pregnancy. Logistic regression analysis showed that people with lower confidence in scientists and self-identified Republicans were more likely to hold beliefs and policy positions that diverged from general scientific consensus.

**Conclusions:**

Overall, we found that a large part of society has embraced beliefs and policy positions not based on general scientific consensus, and that all three issues we examined have become deeply politicized.

Fluoridation of drinking water, milk pasteurization, and acetaminophen use in pregnancy have become among the most visible public health issues in the United States and have been covered extensively by the media since 2025^1-3^. An article on the Johns Hopkins University Bloomberg School of Public Health website declared the debate over acetaminophen use during pregnancy as one of the defining issues in public health in 2025 and made a similar prediction for 2026.^4^ The three health issues not only captured media attention and became linked to the Make American Healthy Again movement, but also entered policy debates at the federal, state, and local levels.^5^ Legislative and regulatory proposals were discussed, introduced, and in some cases turned into policy. These changes happened even though there were no scientific breakthroughs in any of these three areas, and recommendations from public health officials remained unchanged.

Fluoride has been added to public drinking water for decades due to its oral health benefits^6-8^. In 1999, the Centers for Disease Control included water fluoridation on the list of 10 great public health achievements of the 20^th^ century.^9^ Misinformation about water fluoridation has long been present in society. Since the early days of the Cold War, it has been subject to conspiratorial theorizing, but it was generally relegated to the political fringe. However, it has reemerged as an issue and has begun to spread rapidly in recent years. Online posts often cited a discredited scientific study that linked exposure to fluoridated water to lower IQ in children^10^; other studies found no such link.^11,12^ Water fluoridation has become highly politicized, and the discussion has become polarized on social media.^13,14^ In 2025, Florida and Utah, both states led by Republican governors, banned the use of fluoride in public water. According to reports, Florida Governor Ron DeSantis equated adding fluoride to public water to forced medication.^1^ Water fluoridation bans were condemned by the American Dental Association and other health organizations. Similar proposals to ban water fluoridation were made in Ohio and in several counties across the United States but did not pass.^15^ According to one estimate, 420 bills at odds with general scientific consensus on topics including vaccines, milk safety, and water fluoridation were proposed in 2025 across the United States, and approximately 30 were adopted or enacted in 12 states.^16^

Milk has been pasteurized for over a century, which helped reduce foodborne illness and infant mortality. Scientific studies documented the health risks related to drinking raw milk,^17-19^ such as higher likelihood of illness and hospitalization compared to pasteurized milk consumption,^20^ and increased vulnerability to bird flu.^21^ Yet, consumption of raw milk has become increasingly popular, as documented by scientific studies.^22^ Supporters claim that raw milk tastes better, contains more nutrients, and protects against allergies, despite an absence of scientific evidence.^23^ At the same time, the media reported multiple disease outbreaks across the United States that were linked to raw milk consumption.^24^ There were also attempts to change the regulation of the sale of raw milk. Transportation and sale of raw milk from one state to another are prohibited by federal law, but state-level regulations vary. In 2025, Arkansas passed a law allowing the sale of unpasteurized dairy products at farmers' markets.^3^

In Iowa, a bill was proposed to allow the sale of raw milk in grocery stores, which would expand a 2023 law that allowed its sale on dairy farms. The bill was authored by Republican lawmaker Bobby Kaufmann and was supported by Americans for Prosperity, a conservative political advocacy group linked to Charles and David Koch. Iowa Veterinary Medical Association, Iowa Public Health Association, and Iowa Environmental Health Association opposed the bill, emphasizing the public safety risks. The bill was assigned to the Ways and Means Committee, where it passed out of a subcommittee following a 2-1 partisan vote, but ultimately did not become policy.^25^

The use of acetaminophen during pregnancy to reduce fever has long been recommended by governmental agencies and professional medical associations.^26^ Scholars linked untreated fever during pregnancy to a wide range of negative health outcomes in the child.^27,28^ There is no scientific evidence of a causal link between maternal acetaminophen use and neurodevelopmental disorders^29-31^. However, the claims about the negative impacts of acetaminophen use during pregnancy, particularly its potential link to an increasing risk of autism in children, have recently gained traction in society. At a press conference on September 22, 2025, President Donald Trump and Secretary of Health and Human Services Robert F. Kennedy Jr. suggested a link between acetaminophen use and autism. Although acetaminophen was subject to lawsuits in the past alleging a link between its use and autism or other neurodevelopmental disorders, the Trump–Kennedy press conference marked a shift in the issue's prominence by introducing the alleged association into the center of national political discourse. This announcement was criticized by doctors, scientists, and their professional organizations as being unsupported by scientific evidence.^32,33^ Nevertheless, the Food and Drug Administration announced that a process was started to change the safety guidelines for acetaminophen.

We conducted a study to investigate the beliefs about and support for policies regulating water fluoridation, raw milk, and acetaminophen use in the United States. The data came from an original survey fielded online in September and October 2025. The goal was to evaluate the prevalence of beliefs and support for policies regulating these three health-related issues and to identify their predictors. Only a few days before our survey, President Trump linked Tylenol to autism during a press conference, a claim that received widespread media attention. The timing of our survey provided us with a unique opportunity to examine whether attitudes toward an issue that had previously been less politicized exhibit patterns consistent with elite cueing.

Based on the existing scholarship, we focused on the link between the three issues we examined and party identification and confidence in scientists. All three issues have been politicized at the federal and state levels of governance. Given that people take cues from the political parties they identify with, we expected to see partisan differences in their beliefs about these issues as well as in their support for regulation. Previous studies reported a relationship between political partisanship and support for water fluoridation.^34^ We also explored the effect of confidence in scientists. Given general scientific consensus regarding water fluoridation, milk pasteurization, and Tylenol use, we expected that people's attitudes would be related to their confidence in scientists. During the COVID-19 pandemic, scientists recommended COVID-19 vaccination, and research has shown that people with higher trust in scientists and doctors were more likely to receive a vaccination.^35^

## Data and methods

The data came from a national survey conducted online by the authors from September 25 to October 14, 2025. The survey was administered using the Qualtrics platform. Participants were adult residents of the United States recruited by Qualtrics and received financial compensation for completing the survey. Participants provided implied consent electronically before entering the survey. The authors received approval from the Institutional Review Board at Iowa State University before data collection.

The survey included questions measuring beliefs about the safety and regulation of raw milk, fluoridated water, and acetaminophen, party identification, confidence in scientists, as well as age, gender, education, and income. Participants also reported their ZIP code and answered two attention check questions.

Belief about fluoridated water safety was modeled as a 3-point ordinal variable (1 = Safe to drink, 2 = Not sure, 3 = Not safe to drink). Belief about raw milk safety was modeled as a 3-point ordinal variable based on a question asking whether raw milk was as safe to drink as pasteurized milk (1 = Not as safe, 2 = Not sure, 3 = As safe to drink). Belief about the Tylenol–autism link was modeled as a 3-point ordinal variable based on a question asking whether taking Tylenol during pregnancy causes autism in children (1 = No, 2 = Not sure, 3 = Yes). Support for water fluoridation ban was modeled as a 5-point ordinal variable based on a question asking about agreement with a water fluoridation ban (1 = Strongly disagree, 2 = Disagree, 3 = Neither agree nor disagree, 4 = Agree, 5 = Strongly agree). Support for retail sale of raw milk was modeled as a 5-point ordinal variable based on a question asking about agreement with raw milk sale in grocery stores (1 = Strongly disagree, 2 = Disagree, 3 = Neither agree nor disagree, 4 = Agree, 5 = Strongly agree). Support for Tylenol use in pregnancy regulation was modeled as a 5-point ordinal variable based on a question asking about agreement with federal government issuing guidelines against Tylenol use in pregnancy (1 = Strongly disagree, 2 = Disagree, 3 = Neither agree nor disagree, 4 = Agree, 5 = Strongly agree).

Party identification was modeled as a 7-point ordinal variable (1 = Strong Democrat, 2 = Democrat, 3 = Leaning Democrat, 4 = Independent, 5 = Leaning Republican, 6 = Republican, 7 = Strong Republican). Confidence in scientists was modeled as a 4-point ordinal variable (1 = None, 2 = Not too much, 3 = A fair amount, 4 = A great amount). ZIP code data were used to construct a rurality variable based on the Rural-Urban Commuting Area categories.^36^ Data about race and ethnicity were used to create a White ethnicity indicator. We report the full text of survey questions and additional information about variable transformations in the Appendix.

We first described the data and then estimated six regression models using beliefs about the safety and regulation of fluoridated water, raw milk, and Acetaminophen use as dependent variables. Since all dependent variables were ordinal, we estimated ordered logistic regressions. All models included measures of party identification and confidence in scientists, with controls for age, male gender, White ethnicity, education, income, and rurality. We also re-estimated these models using multinomial logistic regression with the middle category as the baseline comparison group. Additionally, we estimated identical models using weighted data (Table S1). Entropy balancing was used to generate weights that matched our data to national benchmarks for age, gender, ethnicity, educational attainment, and party identification.^37^

Participants who failed both attention checks were excluded from the analysis. We used casewise deletion to deal with missing data. We reported adjusted odds ratios with 95% confidence intervals. Goodness-of-fit was assessed with the McFadden's pseudo-*R*^2^ statistic, and multicollinearity was assessed with variance inflation factors. All data management and analysis were conducted in Stata 18.^38^

## Results

The survey included 2135 participants. Approximately 47% of participants were men and the average age was 46.4 years (SD = 17.2). Sixty-four percent of respondents identified as non-Hispanic White, 11% as non-Hispanic Black, 4% as non-Hispanic Asian, 9% as Hispanic, and 11% as multiracial. Fewer than 4% of participants reported not earning a high school diploma, 27% reported earning a high school diploma, 22% reported attending college but not receiving a degree, 14% reported earning an associate's degree, 15% reported earning a college degree, and 20% reported earning a graduate or professional degree. Almost 79% of participants were located in urban areas, 6% in suburban areas, 8% in small towns, and 6% in rural areas. Almost 98% (*n* = 2084) of participants answered at least one of the two attention check questions correctly.

Regarding party identification, 40.1% of respondents identified as Republican, 31.7% as Democrat, 4.1% with another party, and 24.2% as independent. About 41.0% of respondents reported “a great deal” of confidence in scientists, 42.7% “a fair amount” of confidence, 11.8% reported “not too much” confidence, and 4.5% reported having “none.”

Approximately 50.9% of participants thought that fluoridated tap water was safe to drink, 23.0% were not sure, and 26.1% thought it was not safe. Approximately 49.7% of respondents thought that raw milk was not as safe to drink as pasteurized milk, 23.3% were not sure, and 27% of respondents thought it was as safe to drink as pasteurized milk. About 20.5% of respondents thought taking Acetaminophen during pregnancy caused autism, 33.8% were not sure, and 45.7% did not think so. About 2.2% of participants simultaneously believed that fluoridated tap water was not safe to drink, raw milk was as safe to drink as pasteurized milk, and that taking Acetaminophen during pregnancy caused autism.

Approximately 15.5% of participants strongly agreed that fluoridation of tap water should be banned in their state, 19.2% agreed, 41.3% neither agreed nor disagreed (further analysis (Table S5) revealed that female participants and participants with lower educational attainment were more likely to select this category), 14.1% disagreed, and 9.9% strongly disagreed. About 13.1% of respondents strongly agreed that the sale of raw milk should be allowed in their state, 22.5% agreed, 37.7% neither agreed nor disagreed, 14.5% disagreed, and 12.2% strongly disagreed. Approximately 17.1% of participants strongly agreed that government should issue guidelines against women taking Acetaminophen during pregnancy, 23.8% agreed, 30.4% neither agreed nor disagreed, 15.1% disagreed, and 13.6% strongly disagreed. Approximately 5.4% of respondents held the most extreme position (selected “strongly agree”) across all three policy proposals simultaneously.

Across the three safety beliefs (Table 1), confidence in scientists was a consistent and significant predictor. Greater confidence was associated with lower odds of believing that fluoridated water is not safe (aOR = 0.659, 95% CI: 0.590-0.735), lower odds of believing raw milk is as safe as pasteurized milk (aOR = 0.809, 95% CI: 0.728-0.900), and lower odds of endorsing the acetaminophen–autism link (aOR = 0.676, 95% CI: 0.607-0.753).

**Table 1.** Ordered logistic regression on beliefs about fluoridated tap water safety, beliefs about unpasteurized milk safety, and beliefs about acetaminophen–autism link.

	Beliefs about fluoridated tap water safety			Beliefs about unpasteurized milk safety			Beliefs about acetaminophen-autism link		
	aOR	*P*	95% CI	aOR	*P*	95% CI	aOR	*P*	95% CI
Party identification	1.025	0.235	[0.984,1.067]	1.157	<0.001	[1.112,1.203]	1.269	<0.001	[1.219,1.322]
Confidence in scientists	0.659	<0.001	[0.590,0.735]	0.809	<0.001	[0.728,0.900]	0.676	<0.001	[0.607,0.753]
Male gender	0.726	0.001	[0.604,0.872]	1.431	<0.001	[1.198,1.710]	1.413	<0.001	[1.182,1.689]
Age	0.986	<0.001	[0.981,0.992]	0.985	<0.001	[0.980,0.990]	0.996	0.177	[0.991,1.002]
Education	0.817	<0.001	[0.758,0.882]	1.180	<0.001	[1.096,1.271]	1.013	0.737	[0.940,1.092]
White ethnicity	0.773	0.009	[0.637,0.938]	1.079	0.441	[0.890,1.307]	0.908	0.321	[0.749,1.099]
Income	0.926	0.042	[0.860,0.997]	0.982	0.611	[0.914,1.055]	1.061	0.112	[0.986,1.140]
Rurality	0.95	0.294	[0.863,1.046]	1.020	0.685	[0.927,1.122]	0.911	0.056	[0.828,1.003]
Pseudo-*R*^2^	0.061			0.042			0.064		
Observations	2064			2064			2064		

Party identification was significantly associated with beliefs about raw milk safety (aOR = 1.157, 95% CI: 1.112-1.203) and the acetaminophen–autism link (aOR = 1.269, 95% CI: 1.219-1.322), but not with beliefs about fluoridated water safety. Stronger identification with the Democratic Party was associated with higher odds of believing that raw milk is not as safe as pasteurized milk and with higher odds of rejecting the claim that acetaminophen use during pregnancy causes autism.

Older adults had lower odds of believing than younger adults that fluoridated water is not safe to drink (aOR = 0.986 per year, 95% CI: 0.981-0.992), raw milk is as safe to drink as pasteurized milk (aOR = 0.985, 95% CI: 0.980-0.990), and that taking Acetaminophen during pregnancy caused autism (aOR = 0.996, 95% CI: 0.991-1.002). Men had higher odds than women of believing that raw milk is as safe to drink as pasteurized milk (aOR = 1.431, 95% CI: 1.198-1.710), and that taking Acetaminophen during pregnancy caused autism (aOR 1.413, 95% CI: 1.182-1.689), but women had higher odds of believing that fluoridated water is not safe to drink (aOR = 0.726, 95% CI: 0.604-0.872).

Party identification was significantly associated with all three policy outcomes (Table 2). Stronger Republican Party identification was associated with higher odds of supporting a ban on water fluoridation (aOR = 1.177, 95% CI: 1.134-1.222), retail sale of raw milk (aOR = 1.193, 95% CI: 1.149-1.239), and government guidelines against acetaminophen use during pregnancy (aOR = 1.331, 95% CI: 1.281-1.383).

**Table 2.** Ordered logistic regression on support for tap water fluoridation ban, support for unpasteurized milk retail sale, and support for guidelines against acetaminophen use during pregnancy.

	Support for tap water fluoridation ban			Support for unpasteurized milk retail sale			Support for acetaminophen use guidelines		
	aOR	*P*	95% CI	aOR	*P*	95% CI	aOR	*P*	95% CI
Party identification	1.177	<0.001	[1.134,1.222]	1.193	<0.001	[1.149,1.239]	1.331	<0.001	[1.281,1.383]
Confidence in scientists	0.764	<0.001	[0.689,0.848]	0.978	0.675	[0.882,1.084]	0.784	<0.001	[0.708,0.869]
Male gender	1.09	0.322	[0.919,1.292]	1.355	<0.001	[1.143,1.605]	1.348	0.001	[1.138,1.597]
Age	0.975	<0.001	[0.970,0.980]	0.984	<0.001	[0.979,0.989]	0.994	0.019	[0.989,0.999]
Education	1.061	0.101	[0.988,1.139]	1.117	0.002	[1.042,1.198]	1.055	0.127	[0.985,1.131]
White ethnicity	1.033	0.724	[0.862,1.238]	1.019	0.838	[0.851,1.220]	0.89	0.206	[0.744,1.066]
Income	0.989	0.763	[0.924,1.060]	1.057	0.111	[0.987,1.131]	1.066	0.065	[0.996,1.141]
Rurality	1.001	0.991	[0.915,1.094]	1.034	0.466	[0.945,1.131]	0.917	0.056	[0.839,1.002]
Pseudo-*R*^2^	0.038			0.039			0.058		
Observations	2064			2064			2064		

Confidence in scientists was inversely associated with support for banning fluoridation (aOR = 0.764, 95% CI: 0.689-0.848) and with support for issuing guidelines against acetaminophen use (aOR = 0.784, 95% CI: 0.708-0.869), yet it was not significantly associated with support for raw milk retail sale (aOR = 0.978, 95% CI: 0.882-1.084).

Older respondents had lower odds of supporting water fluoridation ban (aOR = 0.975 per year, 95% CI: 0.971-0.980), retail sale of raw milk (aOR = 0.984, 95% CI: 0.979-0.989), and issuing guidelines against acetaminophen use (aOR = 0.994, 95% CI: 0.989-0.999). Male respondents had higher odds of supporting retail sale of raw milk (aOR = 1.355, 95% CI: 1.143-1.605) and guidelines against acetaminophen use during pregnancy (aOR = 1.348, 95% CI: 1.138-1.597), but there was not a significant association with support for water fluoridation ban (aOR = 1.090, 95% CI: 0.919-1.292).

McFadden's pseudo-*R*^2^ values ranged from 0.04 to 0.06 across the six ordered logistic models. The highest variance inflation factor was 2.03, suggesting no evidence of problematic multicollinearity. The results of multinomial logistic regression models (Tables S2-S7) and weighted ordered logistic regression models (Tables S8 and S9) are consistent in terms of magnitude, direction, and statistical significance with the results of our main models.

## Discussion

The results showed a high prevalence of beliefs and support for policies that diverge from general scientific consensus. Sale of raw milk in grocery stores and bans on public water fluoridation have been opposed by many scientists, citing potential negative public health consequences. A quarter of respondents believed that fluoridated tap water was not safe to drink, more than a quarter believed that raw milk was as safe to drink as pasteurized milk, and a fifth thought that taking Acetaminophen during pregnancy could lead to autism. Nevertheless, only 2.2% of participants held all three of these beliefs simultaneously. Approximately 40% of participants supported water fluoridation ban, sale of raw milk in grocery stores, and guidelines against taking Acetaminophen during pregnancy. On the other hand, only 5.4% of participants held the most extreme position on these policy issues and the largest proportion of respondents placed themselves in the middle category, indicating a more neutral position.

Party identification was a statistically significant predictor in five of our six models. Consistently, Republican Party identification was associated with higher odds of expressing beliefs that diverge from general scientific consensus. The observed party differences are consistent with elite cueing theories in political communication. The survey was conducted days after the Trump–Kennedy press conference linking Acetaminophen use during pregnancy to autism, providing a potentially salient cue that may have contributed to the observed partisan differences in beliefs in Acetaminophen safety and regulation. Unlike the other issues, beliefs about the safety of fluoridation were not associated with party identification, suggesting domain-specific politicization dynamics. One possible explanation is that water fluoridation has been a well-established practice across the country since the start of the Cold War, and it has not been subjected to the same degree of politicization as milk pasteurization and Tylenol–autism link.

The results are in line with recent studies showing a partisan gap in science-related attitudes and beliefs.^22^ Journalists have documented how consumption of raw milk products in recent years shifted from the political left to the political right.^39,40^ Our findings underscore the need for further research on the relationship between politics and health. During the COVID-19 pandemic, and the politicization of vaccines, scholars called for the inclusion of political partisanship in public health surveys.^41^ Our results suggest that party identification should also be included in survey-based studies examining attitudes toward raw milk and water fluoridation. For example, many previous studies on attitudes toward fluoride or water fluoridation did not measure political partisanship or ideology^42-44^. Given the increasing politicization of public health, scholars should consider including these questions in their surveys.

The results also showed that confidence in scientists was a statistically significant predictor in five of the six models. The results are in line with scholarship that, during the COVID-19 pandemic, linked trust in science and scientists with more positive attitudes toward COVID-19 vaccination and vaccine uptake.^45^ Opposition to and politicization of science are not new,^46^ but during the recent pandemic, science and scientists were subject to attacks from politicians and misinformation on social media to an unprecedented extent. Trust in scientists decreased in the United States and other countries.^47^ The decrease in trust in scientists might be driven by an affective dislike of scientists as people,^48^ rather than by disagreement with scientific content alone. Trust and confidence in scientists are essential to the social fabric; nevertheless, a recent study showed that strengthening them will be challenging.^49^

We also found that men were more likely than women to believe that taking Acetaminophen during pregnancy could lead to autism and supported issuing guidelines against its use. This gender gap is consistent with findings from opinion polls that showed that men are less likely than women to believe that abortion should be legal in all or most cases.^50^ Differences between men and women regarding Acetaminophen safety and regulation might reflect the presence of subtle paternalistic and sexist attitudes toward women's bodily and reproductive rights.^51^

The results also showed that younger adults were more likely than older adults to hold beliefs and support policies not in line with general scientific consensus. One possible explanation is that the people most animated by the many constituent parts of the Make American Healthy Again movement and are most responsive to their messaging are younger individuals, particularly parents of young children.

Taken together, these findings have broader implications for public health. A large proportion of people with beliefs not consistent with general scientific consensus could have negative effects on society. However, we recognize that individuals' beliefs are legitimate and must be considered regardless of whether they are not rooted in general scientific consensus. The benefits of fluoridated water for dental health have been established, and bans might lead to worsened oral health. The prevalence of these beliefs is a symptom of broader distrust of scientists, public health professionals, and healthcare workers. Professional medical organizations have long voiced their opposition, but many people still hold beliefs and support policies not rooted in general scientific consensus. We have seen this during the COVID-19 pandemic and trust has not recovered since then. An increase in these beliefs will undermine the ability of society to respond successfully to a future health emergency.

The timing of our study enabled us to capture the rapid impact of statements by political leaders on people's attitudes toward health issues. Many participants expressed concerns about its safety, even though the use of Acetaminophen during pregnancy has long been recommended by professional medical associations, and there is no established causal link between Acetaminophen use during pregnancy and the development of neurodevelopmental disorders. Our findings are consistent with the results of a 2026 study that reported a drop in acetaminophen orders in emergency rooms following the September 2025 press conference.^52^

The high degree of politicization of beliefs about raw milk, water fluoridation, and acetaminophen–autism link we reported suggested that public health issues are increasingly considered through a partisan lens. The reliance on partisan cues presents a challenge to messaging from public health leaders and scientists. People may discount scientific guidance when it conflicts with partisan messaging.

The cross-sectional design limited the findings of the study. The statistical results refer to associations and not causal relationships. We encourage scientists to study beliefs that diverge from general scientific consensus over time using panel data. The data were self-reported and it is possible that our study underreported the number of people who hold beliefs or support policies not in line with general scientific consensus due to social desirability bias. Our sample, while closely matching national population on several key parameters, was not nationally representative and oversampled Republican respondents. We addressed this limitation by re-estimating our models with weighted data and the results were substantively identical. The statistical models controlled for several relevant covariates, but the survey did not include questions measuring attitudes toward the Make America Healthy Again movement or Donald Trump. Additionally, the survey did not include questions about the milk sale or tap water regulation in their state, or whether people had community water at home or well water. We encourage scholars to include these questions in future studies. Finally, we note the limitations of the approach we used to determine rurality. While the Rural-Urban Commuting Area classification system is frequently used by scientists due to its precision and economic integration, it might not always correspond with self-perceived identity, particularly in rural areas.

## Supplementary Material

qxag237_Supplementary_Data
