# Supplementary material for: Party identification and public attitudes toward raw milk, water fluoridation, and acetaminophen use
Source: Health Aff Sch. 2026 Sep 24;4(9):qxag237. doi: 10.1093/haschl/qxag237 (PMC13602417; doi:10.1093/haschl/qxag237)
Supplement: Supplementary file 1 [file qxag237_supplementary_data.zip › online appendix.docx]

**Supplementary Appendix**

**Survey Questions**

How old are you? [in years]

What is your home ZIP code? Please enter a five-digit number. [five-digit number]

What is your gender?

- Male/ Female/ Other

What is the highest level of education you have completed?

- Some high school or less; High school diploma; Some college, but no degree; 4 = Associate’s or technical degree; Bachelor’s degree; Graduate or professional degree

What was your total household income before taxes during the past 12 months?

- Less than $25,000; $25,000–$49,999; $50,000–$74,999; $75,000–$99,999; $100,000–$149,999; $150,000 or more; prefer not to say

How much confidence, if any, do you have in each of the following to act in the best interest of the public? Scientists.

- A great deal of confidence; A fair amount of confidence; Not too much confidence; No confidence at all

Generally speaking, do you think of yourself as a Democrat, Republican, an Independent, or something else?

- Republican; Democrat; Independent; Something else

[IF Republican] Would you call yourself a strong Republican or a not very strong Republican?

- Strong Republican; Not a strong Republican

[IF Democrat] Would you call yourself a strong Democrat or a not very strong Democrat?

- Strong Democrat; Not a strong Democrat

[IF Independent OR Something else] Do you think of yourself as closer to the Republican or the Democratic party?

- Republican party; Democratic party; Equally close to both parties

Would you say that fluoridated tap water is generally safe to drink, generally not safe to drink, or are you not sure?

- Safe to drink; Not safe to drink; Not sure

Do you agree or disagree that fluoridation of tap water should be banned in your state?

- Strongly agree; Agree; Neither agree nor disagree; Disagree; Strongly disagree

Would you say that raw (unpasteurized) milk is generally as safe to drink as pasteurized milk, generally not as safe, or are you not sure?

- As safe to drink; Not as safe to drink; Not sure

Do you agree or disagree that the sale of raw milk should be allowed in grocery stores in your state?

- Strongly agree; Agree; Neither agree nor disagree; Disagree; Strongly disagree

Would you say that taking acetaminophen (such as Tylenol) during pregnancy causes autism in children?

- Yes; No; Not sure

Do you agree or disagree that the federal government should issue guidelines against women taking acetaminophen during pregnancy?

- Strongly agree; Agree; Neither agree nor disagree; Disagree; Strongly disagree

Researchers found that participants sometimes answer survey questions without reading them carefully, which could affect data quality. Please select 'Strongly agree' to show that you are paying attention to this question.

- Strongly agree, Somewhat agree, Somewhat disagree, Strongly disagree

We want to check if you're still reading closely. Please select 'very interested'. How interested are you in politics?

- Very interested, Somewhat interested, Not that interested, Not at all interested

**Variable Transformations**

White ethnicity- other categories were merged

Income- “prefer not to say” answers were removed

Party identification- 7-point category variable developed based on the follow-up questions

Rurality- 4-point category variable developed based on the ZIP code data using RUCA classification system

Table S1: Unweighted and weighted sample characteristics

|  | **Unweighted data** | **Weighted data** |
| --- | --- | --- |
| 18 to 24 years | 10.3% | 11.7% |
| 25 to 44 years | 41.3% | 34.5% |
| 45 to 64 years | 29.6% | 30.8% |
| 65 years and older | 18.9% | 22.9% |
| Male | 47.4% | 49.0% |
| Bachelor’s degree or higher | 34.6% | 34.1% |
| White only (non-Hispanic) | 63.9% | 57.8% |
| Republican | 40.0% | 28% |
| Democrat | 31.7% | 28% |

Note: Gender, age, ethnicity, and educational attainment information were obtained from US Census Bureau. Political partisanship information was obtained from Pew Research Center.

**Multinomial Logistic Regression Models**

Table S2: Multinomial logistic regression on beliefs about fluoridated tap water safety (Baseline category= Not sure)

|  | Fluoridated water is safe vs. Not sure | | | Fluoridated water is not safe vs. Not sure | | | |
| --- | --- | --- | --- | --- | --- | --- | --- |
|  | RRR | *p* | 95% CI | RRR | *p* | 95% CI | |
| Party identification | 0.998 | 0.938 | [0.945,1.053] | 1.031 | 0.324 | [0.971,1.094] | |
|  |  |  |  |  |  |  | |
| Confidence in scientists | 1.574 | <0.001 | [1.352,1.832] | 0.918 | 0.267 | [0.788,1.068] | |
|  |  |  |  |  |  |  | |
| Male gender | 1.877 | <0.001 | [1.461,2.412] | 1.350 | 0.032 | [1.027,1.776] | |
|  |  |  |  |  |  |  | |
| Age | 1.002 | 0.683 | [0.994,1.009] | 0.980 | <0.001 | [0.972,0.988] | |
|  |  |  |  |  |  |  | |
| Education | 1.459 | <0.001 | [1.316,1.618] | 1.231 | <0.001 | [1.098,1.381] | |
|  |  |  |  |  |  |  | |
| White ethnicity | 1.400 | 0.013 | [1.074,1.826] | 1.054 | 0.717 | [0.793,1.400] | |
|  |  |  |  |  |  |  | |
| Income | 1.065 | 0.209 | [0.965,1.176] | 0.980 | 0.721 | [0.879,1.094] | |
|  |  |  |  |  |  |  | |
| Rurality | 1.001 | 0.987 | [0.881,1.137] | 0.930 | 0.314 | [0.808,1.071] | |
|  |  | | | | | |  |
| Pseudo r-square | 0.086 | | | | | |  |
| Observations | 2,064 | | | | | |  |

Table S3: Multinomial logistic regression on beliefs about unpasteurized milk safety (Baseline category= Not sure)

|  | Unpasteurized milk is not safe vs. Not sure | | | Unpasteurized milk is safe vs. Not sure | | | |
| --- | --- | --- | --- | --- | --- | --- | --- |
|  | RRR | *p* | 95% CI | RRR | *p* | 95% CI | |
| Party identification | 0.952 | 0.064 | [0.903,1.003] | 1.142 | <0.001 | [1.074,1.215] | |
|  |  |  |  |  |  |  | |
| Confidence in scientists | 1.387 | <0.001 | [1.205,1.595] | 1.086 | 0.324 | [0.922,1.279] | |
|  |  |  |  |  |  |  | |
| Male gender | 1.301 | 0.033 | [1.021,1.657] | 2.357 | <0.001 | [1.778,3.124] | |
|  |  |  |  |  |  |  | |
| Age | 1.006 | 0.113 | [0.999,1.012] | 0.983 | <0.001 | [0.975,0.992] | |
|  |  |  |  |  |  |  | |
| Education | 1.140 | 0.010 | [1.032,1.259] | 1.481 | <0.001 | [1.314,1.669] | |
|  |  |  |  |  |  |  | |
| White ethnicity | 1.144 | 0.298 | [0.888,1.473] | 1.326 | 0.066 | [0.981,1.791] | |
|  |  |  |  |  |  |  | |
| Income | 1.139 | 0.008 | [1.035,1.253] | 1.132 | 0.033 | [1.010,1.270] | |
|  |  |  |  |  |  |  | |
| Rurality | 1.065 | 0.326 | [0.939,1.209] | 1.154 | 0.060 | [0.994,1.340] | |
|  |  | | | | | |  |
| Pseudo r-square | 0.089 | | | | | |  |
| Observations | 2,064 | | | | | |  |

Table S4: Multinomial logistic regression on beliefs about acetaminophen-autism link (Baseline category= Not sure)

|  | No acetaminophen-autism link vs. Not sure | | | Acetaminophen-autism link vs. Not sure | | | |
| --- | --- | --- | --- | --- | --- | --- | --- |
|  | RRR | *p* | 95% CI | RRR | *p* | 95% CI | |
| Party identification | 0.790 | <0.001 | [0.753,0.830] | 1.037 | 0.256 | [0.974,1.103] | |
|  |  |  |  |  |  |  | |
| Confidence in scientists | 1.504 | <0.001 | [1.312,1.723] | 0.862 | 0.065 | [0.736,1.009] | |
|  |  |  |  |  |  |  | |
| Male gender | 0.838 | 0.118 | [0.672,1.046] | 1.411 | 0.014 | [1.072,1.856] | |
|  |  |  |  |  |  |  | |
| Age | 0.989 | 0.001 | [0.982,0.995] | 0.975 | <0.001 | [0.967,0.984] | |
|  |  |  |  |  |  |  | |
| Education | 1.183 | <0.001 | [1.080,1.296] | 1.284 | <0.001 | [1.144,1.441] | |
|  |  |  |  |  |  |  | |
| White ethnicity | 1.417 | 0.004 | [1.118,1.795] | 1.389 | 0.028 | [1.036,1.863] | |
|  |  |  |  |  |  |  | |
| Income | 1.021 | 0.641 | [0.935,1.116] | 1.158 | 0.011 | [1.034,1.297] | |
|  |  |  |  |  |  |  | |
| Rurality | 1.041 | 0.497 | [0.927,1.168] | 0.911 | 0.238 | [0.779,1.064] | |
|  |  | | | | | |  |
| Pseudo r-square | 0.095 | | | | | |  |
| Observations | 2,064 | | | | | |  |

Table S5: Multinomial logistic regression on support for tap water fluoridation ban (Baseline category= Neither agree nor disagree)

|  | Strongly disagree vs. neither agree nor disagree | | | Disagree vs. neither agree nor disagree | | | Agree vs. neither agree nor disagree | | | Strongly agree vs. neither agree nor disagree | | |
| --- | --- | --- | --- | --- | --- | --- | --- | --- | --- | --- | --- | --- |
|  | RRR | *p* | 95% CI | RRR | *p* | 95% CI | RRR | *p* | 95% CI | RRR | *p* | 95% CI |
| Party identification | 0.833 | <0.001 | [0.774,0.895] | 0.923 | 0.011 | [0.867,0.982] | 1.067 | 0.027 | [1.007,1.131] | 1.126 | <0.001 | [1.056,1.200] |
|  |  |  |  |  |  |  |  |  |  |  |  |  |
| Confidence in scientists | 2.065 | <0.001 | [1.594,2.676] | 1.323 | 0.004 | [1.093,1.602] | 1.012 | 0.884 | [0.864,1.184] | 0.885 | 0.155 | [0.748,1.047] |
|  |  |  |  |  |  |  |  |  |  |  |  |  |
| Male gender | 2.127 | <0.001 | [1.512,2.992] | 1.333 | 0.054 | [0.996,1.786] | 1.617 | <0.001 | [1.241,2.105] | 1.655 | 0.001 | [1.241,2.207] |
|  |  |  |  |  |  |  |  |  |  |  |  |  |
| Age | 1.023 | <0.001 | [1.012,1.033] | 1.015 | 0.001 | [1.006,1.024] | 0.974 | <0.001 | [0.965,0.982] | 0.978 | <0.001 | [0.969,0.987] |
|  |  |  |  |  |  |  |  |  |  |  |  |  |
| Education | 1.206 | 0.007 | [1.052,1.382] | 1.130 | 0.044 | [1.003,1.272] | 1.220 | 0.001 | [1.090,1.365] | 1.255 | <0.001 | [1.111,1.418] |
|  |  |  |  |  |  |  |  |  |  |  |  |  |
| White ethnicity | 1.603 | 0.013 | [1.104,2.329] | 1.085 | 0.606 | [0.795,1.482] | 1.163 | 0.289 | [0.879,1.539] | 1.320 | 0.078 | [0.969,1.798] |
|  |  |  |  |  |  |  |  |  |  |  |  |  |
| Income | 1.028 | 0.678 | [0.901,1.175] | 1.198 | 0.002 | [1.068,1.344] | 1.047 | 0.405 | [0.940,1.167] | 1.105 | 0.100 | [0.981,1.244] |
|  |  |  |  |  |  |  |  |  |  |  |  |  |
| Rurality | 0.892 | 0.250 | [0.735,1.084] | 0.903 | 0.224 | [0.766,1.064] | 0.995 | 0.947 | [0.865,1.145] | 0.909 | 0.243 | [0.774,1.067] |
|  |  |  |  |  |  |  |  |  |  |  |  |  |
| Pseudo r-square | 0.070 | | | | | | | | | | | |
| Observations | 2,064 | | | | | | | | | | | |

Table S6: Multinomial logistic regression on support for unpasteurized milk retail sale (Baseline category= Neither agree nor disagree)

|  | Strongly disagree vs. neither agree nor disagree | | | Disagree vs. neither agree nor disagree | | | Agree vs. neither agree nor disagree | | | Strongly agree vs. neither agree nor disagree | | |
| --- | --- | --- | --- | --- | --- | --- | --- | --- | --- | --- | --- | --- |
|  | RRR | *p* | 95% CI | RRR | *p* | 95% CI | RRR | *p* | 95% CI | RRR | *p* | 95% CI |
| Party identification | 0.854 | <0.001 | [0.797,0.914] | 0.959 | 0.187 | [0.902,1.021] | 1.113 | <0.001 | [1.052,1.177] | 1.190 | <0.001 | [1.110,1.275] |
|  |  |  |  |  |  |  |  |  |  |  |  |  |
| Confidence in scientists | 1.274 | 0.016 | [1.045,1.552] | 1.117 | 0.220 | [0.936,1.334] | 1.08 | 0.330 | [0.925,1.261] | 1.127 | 0.225 | [0.929,1.366] |
|  |  |  |  |  |  |  |  |  |  |  |  |  |
| Male gender | 1.213 | 0.220 | [0.891,1.650] | 1.138 | 0.379 | [0.853,1.518] | 1.571 | 0.001 | [1.215,2.031] | 2.102 | <0.001 | [1.529,2.890] |
|  |  |  |  |  |  |  |  |  |  |  |  |  |
| Age | 1.000 | 0.971 | [0.991,1.009] | 1.002 | 0.639 | [0.994,1.010] | 0.974 | <0.001 | [0.966,0.982] | 0.977 | <0.001 | [0.967,0.987] |
|  |  |  |  |  |  |  |  |  |  |  |  |  |
| Education | 1.221 | 0.002 | [1.077,1.385] | 1.155 | 0.016 | [1.027,1.300] | 1.303 | <0.001 | [1.167,1.454] | 1.446 | <0.001 | [1.263,1.657] |
|  |  |  |  |  |  |  |  |  |  |  |  |  |
| White ethnicity | 1.220 | 0.232 | [0.880,1.691] | 1.181 | 0.293 | [0.866,1.609] | 1.139 | 0.353 | [0.865,1.500] | 1.259 | 0.183 | [0.897,1.768] |
|  |  |  |  |  |  |  |  |  |  |  |  |  |
| Income | 0.956 | 0.469 | [0.848,1.079] | 1.096 | 0.113 | [0.979,1.228] | 1.088 | 0.115 | [0.980,1.209] | 1.083 | 0.228 | [0.951,1.234] |
|  |  |  |  |  |  |  |  |  |  |  |  |  |
| Rurality | 1.035 | 0.680 | [0.878,1.221] | 1.001 | 0.989 | [0.856,1.171] | 1.101 | 0.172 | [0.959,1.264] | 1.105 | 0.251 | [0.932,1.311] |
|  |  |  |  |  |  |  |  |  |  |  |  |  |
| Pseudo r-square | 0.064 | | | | | | | | | | | |
| Observations | 2,064 | | | | | | | | | | | |

Table S7: Multinomial logistic regression on support for guidelines against acetaminophen use during pregnancy (Baseline category= Neither agree nor disagree)

|  | Strongly disagree vs. neither agree nor disagree | | | Disagree vs. neither agree nor disagree | | | Agree vs. neither agree nor disagree | | | Strongly agree vs. neither agree nor disagree | | |
| --- | --- | --- | --- | --- | --- | --- | --- | --- | --- | --- | --- | --- |
|  | RRR | *p* | 95% CI | RRR | *p* | 95% CI | RRR | *p* | 95% CI | RRR | *p* | 95% CI |
| Party identification | 0.690 | <0.001 | [0.639,0.744] | 0.872 | <0.001 | [0.817,0.930] | 1.119 | <0.001 | [1.056,1.187] | 1.165 | <0.001 | [1.090,1.244] |
|  |  |  |  |  |  |  |  |  |  |  |  |  |
| Confidence in scientists | 1.910 | <0.001 | [1.526,2.389] | 1.293 | 0.007 | [1.074,1.558] | 0.945 | 0.468 | [0.811,1.101] | 1.071 | 0.436 | [0.901,1.273] |
|  |  |  |  |  |  |  |  |  |  |  |  |  |
| Male gender | 0.790 | 0.150 | [0.573,1.089] | 0.741 | 0.050 | [0.549,1.001] | 1.308 | 0.043 | [1.008,1.698] | 1.194 | 0.230 | [0.894,1.596] |
|  |  |  |  |  |  |  |  |  |  |  |  |  |
| Age | 0.996 | 0.355 | [0.987,1.005] | 1.007 | 0.122 | [0.998,1.016] | 0.996 | 0.263 | [0.988,1.003] | 0.988 | 0.007 | [0.979,0.997] |
|  |  |  |  |  |  |  |  |  |  |  |  |  |
| Education | 1.224 | 0.003 | [1.073,1.396] | 1.174 | 0.010 | [1.039,1.327] | 1.231 | <0.001 | [1.103,1.373] | 1.299 | <0.001 | [1.149,1.469] |
|  |  |  |  |  |  |  |  |  |  |  |  |  |
| White ethnicity | 1.775 | 0.001 | [1.270,2.480] | 1.245 | 0.173 | [0.908,1.706] | 1.369 | 0.030 | [1.032,1.817] | 1.177 | 0.304 | [0.863,1.605] |
|  |  |  |  |  |  |  |  |  |  |  |  |  |
| Income | 0.931 | 0.261 | [0.821,1.055] | 1.098 | 0.118 | [0.977,1.235] | 1.108 | 0.059 | [0.996,1.233] | 1.073 | 0.245 | [0.953,1.209] |
|  |  |  |  |  |  |  |  |  |  |  |  |  |
| Rurality | 1.002 | 0.978 | [0.847,1.187] | 0.981 | 0.811 | [0.841,1.146] | 0.878 | 0.071 | [0.762,1.011] | 0.891 | 0.156 | [0.759,1.045] |
|  |  |  |  |  |  |  |  |  |  |  |  |  |
| Pseudo r-square | 0.080 | | | | | | | | | | | |
| Observations | 2,064 | | | | | | | | | | | |

. **Weighted Models**

Table S8: Weighted ordered logistic regression on beliefs about fluoridated tap water safety, beliefs about unpasteurized milk safety, and beliefs about acetaminophen-autism link

|  | Beliefs about fluoridated tap water safety | | | Beliefs about unpasteurized milk safety | | | Beliefs about acetaminophen-autism link | | |
| --- | --- | --- | --- | --- | --- | --- | --- | --- | --- |
|  | aOR | *p* | 95% CI | aOR | *p* | 95% CI | aOR | *p* | 95% CI |
| Party identification | 1.020 | 0.420 | [0.973,1.069] | 1.152 | <0.001 | [1.099,1.207] | 1.256 | <0.001 | [1.197,1.319] |
|  |  |  |  |  |  |  |  |  |  |
| Confidence in scientists | 0.672 | <0.001 | [0.590,0.766] | 0.850 | 0.006 | [0.758,0.954] | 0.678 | <0.001 | [0.600,0.765] |
|  |  |  |  |  |  |  |  |  |  |
| Male gender | 0.719 | 0.003 | [0.578,0.894] | 1.373 | 0.002 | [1.129,1.671] | 1.489 | <0.001 | [1.223,1.814] |
|  |  |  |  |  |  |  |  |  |  |
| Age | 0.987 | <0.001 | [0.981,0.993] | 0.984 | <0.001 | [0.979,0.990] | 0.995 | 0.084 | [0.989,1.001] |
|  |  |  |  |  |  |  |  |  |  |
| Education | 0.858 | 0.001 | [0.783,0.939] | 1.167 | <0.001 | [1.074,1.268] | 1.025 | 0.567 | [0.941,1.117] |
|  |  |  |  |  |  |  |  |  |  |
| White ethnicity | 0.732 | 0.007 | [0.585,0.917] | 1.062 | 0.591 | [0.853,1.322] | 0.922 | 0.455 | [0.745,1.141] |
|  |  |  |  |  |  |  |  |  |  |
| Income | 0.902 | 0.023 | [0.826,0.986] | 0.973 | 0.507 | [0.898,1.055] | 1.027 | 0.516 | [0.947,1.114] |
|  |  |  |  |  |  |  |  |  |  |
| Rurality | 0.990 | 0.849 | [0.888,1.102] | 1.006 | 0.912 | [0.903,1.121] | 0.935 | 0.205 | [0.842,1.038] |
|  |  |  |  |  |  |  |  |  |  |
| Pseudo r-square | 0.057 | | | 0.039 | | | 0.062 | | |
| Observations | 2,064 | | | 2,064 | | | 2,064 | | |

Table S9: Weighted ordered logistic regression on support for tap water fluoridation ban, support for unpasteurized milk retail sale, and support for guidelines against acetaminophen use during pregnancy

|  | Support for tap water fluoridation ban | | | Support for unpasteurized milk retail sale | | | Support for acetaminophen use guidelines | | |
| --- | --- | --- | --- | --- | --- | --- | --- | --- | --- |
|  | aOR | *p* | 95% CI | aOR | *p* | 95% CI | aOR | *p* | 95% CI |
| Party identification | 1.165 | <0.001 | [1.113,1.219] | 1.178 | <0.001 | [1.124,1.235] | 1.312 | <0.001 | [1.252,1.375] |
|  |  |  |  |  |  |  |  |  |  |
| Confidence in scientists | 0.808 | 0.001 | [0.714,0.913] | 0.985 | 0.808 | [0.874,1.110] | 0.803 | <0.001 | [0.712,0.905] |
|  |  |  |  |  |  |  |  |  |  |
| Male gender | 1.110 | 0.303 | [0.910,1.356] | 1.415 | 0.001 | [1.162,1.721] | 1.395 | 0.001 | [1.140,1.707] |
|  |  |  |  |  |  |  |  |  |  |
| Age | 0.974 | <0.001 | [0.968,0.980] | 0.982 | <0.001 | [0.976,0.987] | 0.990 | <0.001 | [0.984,0.995] |
|  |  |  |  |  |  |  |  |  |  |
| Education | 1.071 | 0.107 | [0.985,1.165] | 1.104 | 0.011 | [1.023,1.192] | 1.066 | 0.103 | [0.987,1.151] |
|  |  |  |  |  |  |  |  |  |  |
| White ethnicity | 0.979 | 0.846 | [0.793,1.209] | 1.032 | 0.772 | [0.835,1.275] | 0.904 | 0.344 | [0.733,1.114] |
|  |  |  |  |  |  |  |  |  |  |
| Income | 0.967 | 0.430 | [0.891,1.050] | 1.023 | 0.564 | [0.947,1.106] | 1.017 | 0.666 | [0.940,1.101] |
|  |  |  |  |  |  |  |  |  |  |
| Rurality | 1.027 | 0.593 | [0.931,1.132] | 1.060 | 0.254 | [0.959,1.170] | 0.936 | 0.204 | [0.846,1.036] |
|  |  |  |  |  |  |  |  |  |  |
| Pseudo r-square | 0.035 | | | 0.037 | | | 0.054 | | |
| Observations | 2,064 | | | 2,064 | | | 2,064 | | |
